# Strategies to reduce the risk of SARS-CoV-2 importation from international travellers: modelling estimations for the United Kingdom, July 2020

**DOI:** 10.2807/1560-7917.ES.2021.26.39.2001440

**Published:** 2021-09-30

**Authors:** Samuel Clifford, Billy J Quilty, Timothy W Russell, Yang Liu, Yung-Wai D Chan, Carl A B Pearson, Rosalind M Eggo, Akira Endo, CMMID COVID-19 Working Group, Stefan Flasche, W John Edmunds, Centre for Mathematical Modelling of Infectious Diseases (CMMID) COVID-19 Working Group, Centre for Mathematical Modelling of Infectious Diseases (CMMID) COVID-19 Working Group, Katharine Sherratt, Stéphane Hué, Matthew Quaife, Nikos I Bosse, Graham Medley, Megan Auzenbergs, Adam J Kucharski, Nicholas G Davies, Oliver Brady, Sophie R Meakin, Rein M G J Houben, Katherine E Atkins, Kiesha Prem, C Julian Villabona-Arenas, Hamish P Gibbs, Thibaut Jombart, Charlie Diamond, Petra Klepac, Arminder K Deol, Rachel Lowe, James W Rudge, Mark Jit, Sebastian Funk, Gwenan M. Knight, Simon R. Procter, David Simons, Quentin J Leclerc, James D Munday, Amy Gimma, Georgia R Gore-Langton, Christopher I Jarvis, Jon C Emery, Anna M Foss, Kathleen O'Reilly, Joel Hellewell, Emily S Nightingale, Kevin van Zandvoort, Damien C Tully, Sam Abbott, Kaja Abbas, Fiona Yueqian Sun, Alicia Rosello

**Affiliations:** 1 Centre for Mathematical Modelling of Infectious Diseases, Department of Infectious Disease Epidemiology, London School of Hygiene and Tropical Medicine, London, United Kingdom; 2 The members of the group are listed under Investigators

**Keywords:** SARS-CoV-2, COVID-19, travel screening, quarantine, PCR testing

## Abstract

**Background:**

To mitigate SARS-CoV-2 transmission risks from international air travellers, many countries implemented a combination of up to 14 days of self-quarantine upon arrival plus PCR testing in the early stages of the COVID-19 pandemic in 2020.

**Aim:**

To assess the effectiveness of quarantine and testing of international travellers to reduce risk of onward SARS-CoV-2 transmission into a destination country in the pre-COVID-19 vaccination era.

**Methods:**

We used a simulation model of air travellers arriving in the United Kingdom from the European Union or the United States, incorporating timing of infection stages while varying quarantine duration and timing and number of PCR tests.

**Results:**

Quarantine upon arrival with a PCR test on day 7 plus a 1-day delay for results can reduce the number of infectious arriving travellers released into the community by a median 94% (95% uncertainty interval (UI): 89–98) compared with a no quarantine/no test scenario. This reduction is similar to that achieved by a 14-day quarantine period (median > 99%; 95% UI: 98–100). Even shorter quarantine periods can prevent a substantial amount of transmission; all strategies in which travellers spend at least 5 days (mean incubation period) in quarantine and have at least one negative test before release are highly effective (median reduction 89%; 95% UI: 83–95)).

**Conclusion:**

The effect of different screening strategies impacts asymptomatic and symptomatic individuals differently. The choice of an optimal quarantine and testing strategy for unvaccinated air travellers may vary based on the number of possible imported infections relative to domestic incidence.

## Background

Severe acute respiratory syndrome coronavirus 2 (SARS-CoV-2), the causative agent of coronavirus disease (COVID-19), emerged in Wuhan, China in late 2019 and was rapidly disseminated globally through international air travel in the first half of 2020 [1]. In addition to non-pharmaceutical interventions (NPIs) to reduce domestic transmission, many countries implemented restrictions on incoming international travel such as mandatory quarantine, testing and travel bans, with the aim of preventing or reducing further importation and onward transmission [2]. 

During this early period of the COVID-19 pandemic prior to the roll-out of vaccines in late 2020, a number of countries in Europe and the Asia Pacific region implemented a mandatory quarantine upon arrival, which typically had a duration of 14 days [2,3]. It is expected that, by day 14, at least 95% of all infected individuals who will become symptomatic have done so [4]. However, the median incubation period for SARS-CoV-2 is ca 5 days (95% confidence interval: 4.1 to 7.0) [4] and, assuming that travellers are equally likely to travel at any point in this period, a 5-day quarantine on arrival should suffice to allow more than 50% of the infected travellers to become symptomatic and be managed accordingly. Quarantine, either at home or at managed facilities [5], may lead to negative psychological effects stemming from social isolation [6,7] and financial stress [8]. Hence, there is considerable interest in reducing the period of quarantine, assuming it is safe to do so.

In addition to quarantine, several countries introduced a requirement for travellers to undergo testing for SARS-CoV-2 infection with RT-PCR (hereafter PCR). Such testing is commonly performed by taking nasopharyngeal or throat swabs of individuals and analysing the resulting sample for the presence of SARS-CoV-2 RNA [9]. PCR screening may be conducted before the flight and/or after arrival to allow detection of infected travellers. In some countries, testing is also used to reduce or eliminate quarantine for travellers without a confirmed infection. For example, in the summer of 2020, Japan allowed business travellers from designated low-risk countries to bypass the 14-day quarantine period given a negative PCR test result upon arrival [10].

Here we investigated the effectiveness of several strategies available in the pre-vaccination era of the SARS-CoV-2 pandemic to reduce the number of arriving infectious travellers as well as the potential for transmission in the community. We assessed the impact of varying the duration of quarantine and the timing and number of PCR tests, as well as the prevalence in and travel volume from the European Union (EU) and the United States (US) to the United Kingdom (UK) as of July 2020, while also accounting for the natural history of SARS-CoV-2 infection. 

## Methods

### Travel screening trajectories

**Figure 1:**
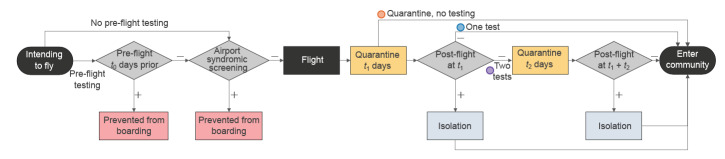
Possible traveller trajectories for the considered SARS-CoV-2 screening scenarios pre- and post-flight

The possible SARS-CoV-2 screening outcomes for air travellers are as follows: (i) prevented from travelling following detection of SARS-CoV-2 infection either through syndromic screening at the airport or a positive pre-flight PCR test, (ii) released after the mandatory isolation period following detection of SARS-CoV-2 infection either by a positive PCR test upon entry or a follow-up positive PCR test after a negative result upon entry, (iii) released after a second negative test during the quarantine period, and (iv) in the absence of post-entry testing, travellers will be released after the mandatory quarantine period (which, in the model, may have a duration of 0 days) (Figure 1).

SARS-CoV-2: severe acute respiratory syndrome coronavirus 2.

Testing for SARS-CoV-2 infection (grey diamonds) may be conducted pre-flight and/or post-flight and may occur alongside quarantine periods (yellow boxes). Travellers who are found to be infected pre-flight are prevented from boarding (pink boxes); travellers found to be infected during quarantine are diverted to isolation (light blue boxes). Travellers enter the community after the required number of negative tests (regardless of infection status) or after finishing their allocated duration of quarantine or isolation. Coloured circles indicate the number of tests along that trajectory.

### Estimating the number of infected travellers

We simulated the number of infected air travellers intending to fly to a destination country in a given week based on the monthly volume of flights between the origin and destination, and considering the prevalence of COVID-19 in the origin country (Supplementary Table S1). We used the UK as a case study for the destination country. We assumed that the inbound and outbound travel is balanced on average. To estimate the number of people travelling into the UK, we halved the total number of monthly traveller movements.

The time of each intending traveller’s flight was sampled uniformly between the time of exposure to SARS-CoV-2 and time of recovery. We modelled international travellers coming either from the US or the EU, using publicly available Civil Aviation Authority data for April and May 2020 [11,12]. Estimates of current COVID-19 infection prevalence were derived from reported cases and death time series data while adjusting for reporting delays and under-reporting based on case-fatality ratio estimates [13,14]. EU-wide prevalence was calculated as a population-weighted mean of available country-level estimates of the non-UK EU countries (except Malta, for which a prevalence estimate was not available).

For each simulation, we sampled the number of weekly intending travellers, the proportion of those who were infected, and the proportion of infected travellers who were symptomatic and asymptomatic [15] (details are provided in Supplementary Table S1).

### Risk mitigation strategies

We considered several risk mitigation strategies. Travellers are subjected to a quarantine which lasts either (i) 0 days (low stringency); (ii) 3, 5, 7 or 9 days (moderate stringency); or (iii) 14 days (high/maximum stringency) (Supplementary Table S2). In the low and moderate stringency levels, travellers may also be tested on the final day of their quarantine and wait an additional day for their results [16]; in the low stringency setting, this effectively enforces a 1-day quarantine. For the high stringency scenario, travellers are assumed to undergo two stages of PCR testing; if they receive two negative tests during their post-arrival quarantine period, they are cleared to leave quarantine early (i.e. the day after their final test to account for test delays). Travellers who become symptomatic during their quarantine period must meet all of the following conditions for release: (i) they must no longer display symptoms, (ii) it must be at least 7 days since the onset of symptoms, and (iii) they must have been in quarantine for at least 14 days [3].

### Model assumptions

We assumed that syndromic screening is performed before departure, which may consist of thermal scanning and/or monitoring of symptoms such as cough and fever [17]. Given the awareness of the pandemic and guidance issued on travelling while ill, we assumed in all scenarios that 70% of currently-symptomatic travellers do not fly (as modelled by Gostic et al. [18,19]).

Pre-flight PCR testing was required by some countries and airlines. In July 2020, the International Air Transport Association recommended testing within 24 h of departure [20] but some countries required testing within 7 days of the flight [21]. Considering this wide range of pre-travel test recommendations, we chose to include a 4-day pre-flight test as a midpoint.

### Case definitions and detection of infected travellers

We defined a symptomatic infection as an individual whose symptoms e.g. fever, cough, loss of sense of taste or smell, would be detectable by the individual, airport staff, quarantine staff, or a healthcare worker and typically lead to self-isolation, consistent with that defined by the UK’s National Health Service [22]. We defined an asymptomatic infection as one where the individual never develops symptoms throughout the duration of their infection, according to Buitrago-Garcia et al. [15]. We assumed that the sensitivity of PCR testing for a nasopharyngeal or throat swab varies over the course of infection, peaking around onset of symptoms [23], and that test specificity is 100% [24]. We assumed that the probability of detecting an asymptomatic infection through PCR testing is 0.62 times that of a symptomatic infection, as reported by Chau et al. [25] for nasopharyngeal or throat swab samples collected from quarantining travellers (Supplementary Table S3). We derived the proportion of asymptomatic travellers by quantile, matching the 95% prediction interval (0.03–0.55) of Buitrago-Garcia et al. as a beta distribution, giving a median of 0.21 (i.e. 21% of travellers being asymptomatic on average across model simulations) [15].

The duration of the incubation period (time from exposure to onset of symptoms, and assumed here to also represent the timing of peak probability of detection in both symptomatic and asymptomatic individuals) was taken from Lauer et al. [26] (Supplementary Table S3). The duration of the latent period (time from exposure to the onset of infectiousness) [27], was derived from Ashcroft et al. (corrected version of He et al.), and was also assumed to be equal for symptomatic and asymptomatic individuals [28,29]. The duration of the infectious period of symptomatic cases was derived from Wolfel et al. [30], while that of asymptomatic cases was derived from Byrne et al. [31], with asymptomatic cases being infectious for a shorter period than symptomatic cases (median: 5.2 vs 7.1 days) (Supplementary Table S3). 

Given the natural history of infection parameters, we estimated the number of infected travellers entering the community in each scenario who would have the potential to cause onward transmission, i.e. those still in their infectious or pre-infectious period. In addition, we calculated the number of infectious days spent in the community for each infected traveller following their release. These values were then summed for all individuals to give the total person-days of infectiousness spent in the community for each scenario. We report these values for the estimated weekly travellers based on travel volumes and per 10,000 infected travellers, with 1,000 bootstrap replications each to generate medians and 95% and 50% uncertainty intervals (UI). We calculated rate ratios (RR) in each screening scenario for the number of infectious individuals released and infectious days remaining compared to the low scenario (syndromic screening, no quarantine, and no PCR testing) and maximum scenario (14-day quarantine, no testing) were calculated with 10,000 travellers per simulation to avoid small number biases and were bootstrapped 1,000 times to generate medians and 95% and 50% UI. All analysis was conducted in R version 4.0.2 [32] and the code is available https://github.com/cmmid/pcr_entry_screening_eurosurv.

## Results

Based on the prevalence of COVID-19 in the respective countries on 20 July 2020, we estimated that the expected proportion of travellers who entered the UK while infectious was substantially higher for flights originating in the US than for those originating in the EU (Supplementary Figure S3). However, as the prevalence of COVID-19 in the US was ca 14 times that in the EU in July 2020 and travel volumes were ca 8 times lower than those from the EU, we expect approximately half the number of infectious travellers arriving from the EU than from the US (Supplementary Table S1). Here we focus on the estimates for travel from the US and provide results for travel from the EU for comparison in the Supplement (Figure S1 and S2).

### Effectiveness of quarantine and testing

**Figure 2:**
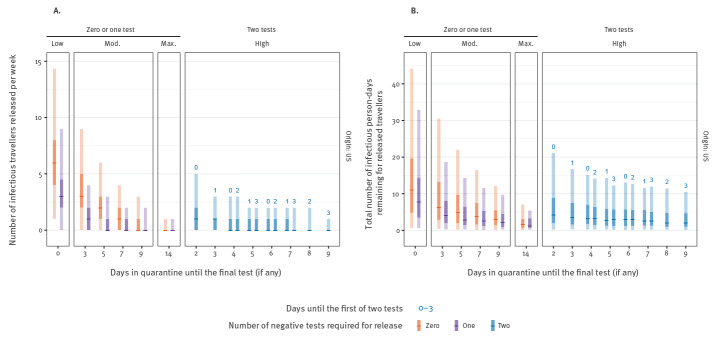
Expected number of infectious and pre-infectious individuals entering the United Kingdom from the United States (A) and total infectious person-days remaining after release (B) based on estimated travel volumes and quarantine duration with no pre-flight testing, United Kingdom, July 2020

As a baseline for comparison, we used the lowest stringency scenario considered i.e. 70% of currently symptomatic travellers are prevented from boarding, but no quarantine or testing is conducted. In this scenario, a median of six infectious travellers (95% UI: 1–14.2) would enter the community from the US per week (Figure 2A). By introducing a mandatory quarantine period of 7 days, this can be reduced to one infectious traveller (95% UI: 0–4), preventing ca 80% of infectious travellers from entering the community (RR: 0.17; 95% UI: 0.10–0.26). A mandatory quarantine period of 14 days resulted in zero to one infectious entry per week, almost fully preventing importation (RR: 0.02; 95% UI: 0.00–0.03).

Test results are assumed to be delayed by 1 day and individuals leave quarantine 1 day after their final test. Central dash: median; light bar: 95% uncertainty interval; dark bar: 50% uncertainty interval.

**Figure 3:**
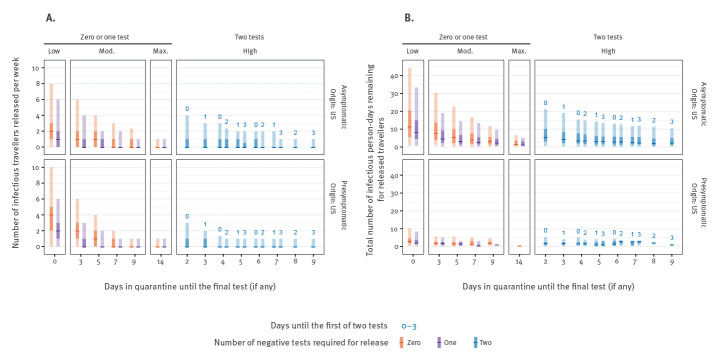
Expected number of infectious and pre-infectious individuals entering the United Kingdom from the United States (A) and total infectious person-days remaining after release (B) based on estimated travel volumes and quarantine duration with no pre-flight testing, stratified by asymptomatic or pre-symptomatic infection, United Kingdom, July 2020

Longer quarantine periods increase the fraction of pre-symptomatic infected travellers who would have their onset of symptoms during the quarantine and hence self-isolate until symptoms subside (Figure 2A, Figure 3A). Accordingly, we estimated a more pronounced impact of interventions targeting travellers on the number of infectious person-days from travellers, particularly for those who would eventually become symptomatic (Figure 2B, Figure 3B). The uncertainty in the number of remaining infectious person-days is driven by variability in the detection of asymptomatic infections, as they will never be detected by pre-flight syndromic screening, are less likely to be detected by PCR and will never develop symptoms that trigger mandatory isolation.

Max: maximum; Mod: moderate; US: United States.

Test results are assumed to be delayed by 1 day and individuals leave quarantine 1 day after their final test. Central dash: median; light bar: 95% uncertainty interval; dark bar: 50% uncertainty interval.

**Figure 4:**
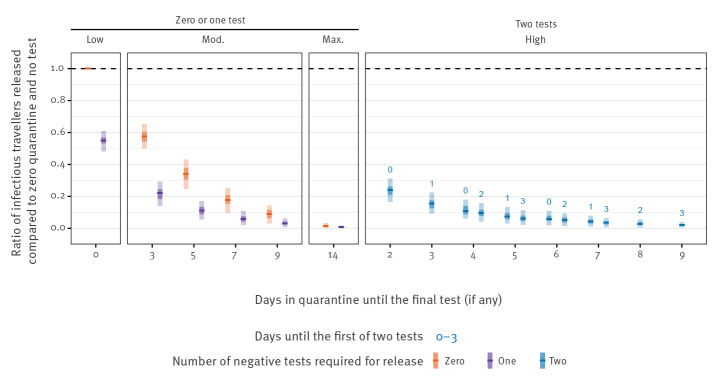
Risk reduction per infected traveller compared to a baseline of syndromic screening and no quarantine and no testing on arrival, United Kingdom, July 2020

Conducting a single test for all travellers at the end of the described quarantine periods further reduced the median number of infectious entering travellers from the US, with an RR of 0.55 (95% UI: 0.48–0.61) for a test on arrival, with release on day 1; an RR of 0.11 (95% UI: 0.05–0.17) for a test on day 5, release on day 6; an RR of 0.06 (95% UI: 0.02–0.11) for a test on day 7, release on day 8; an RR of 0.03 (95% UI: 0.00–0.06) for a test on day 9 with release on day 10; and an RR of 0.01 (95% UI: 0.00–0.02) for a test on day 14, release on day 15, when compared with the lowest stringency scenario (Figure 4). Requiring a second round of testing had marginal impact, although a quarantine period of 9 days with two tests and early exit may be able to largely replicate the impact of a 14-day quarantine period (RR: 0.02; 95% UI: 0.00–0.04).

Max: maximum; Mod: moderate.

Baseline scenario is indicated by the left-most value (Low, 0 days quarantine, no testing). We assumed that test results are delayed by 1 day and individuals leave quarantine 1 day after their final test. Central dash: median; light bar: 95% uncertainty interval; dark bar: 50% uncertainty interval. Product of 1,000 infected arriving travellers and 1,000 simulations per scenario.

### Rate ratios by symptom status

**Figure 5:**
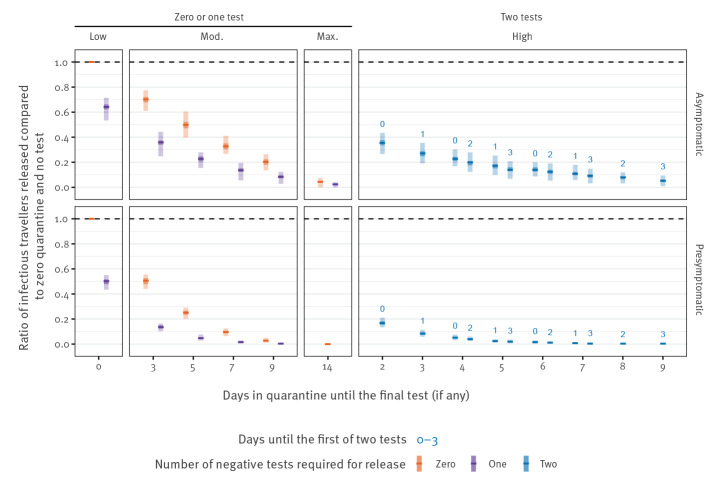
Risk reduction per infected traveller compared to a baseline of syndromic screening and no quarantine and no testing on arrival, stratified by asymptomatic or pre-symptomatic infection, United Kingdom, July 2020

We stratified the above RR by whether the infection is asymptomatic or pre-symptomatic. We observed that the strategies are more effective against those with pre-symptomatic than asymptomatic infections (Figure 5). The introduction of a test on arrival (Figure 5, low, 1 day) reduces the number of asymptomatic entering travellers by 36% (95% UI: 28–47) and pre-symptomatic by 50% (95% UI: 45–56), beyond that which is captured by syndromic screening alone (Figure 5, low, 0 days). At maximum stringency, a 14-day quarantine period is able to reduce the number of symptomatic entering travellers by more than 99% (95% UI: 99–100) and asymptomatic entering travellers by 96% (95% UI: 93–100).

Max: maximum; Mod: moderate.

Baseline scenario is indicated by the left-most value (Low, 0 days quarantine, no testing). We assumed that test results are delayed by 1 day and individuals leave quarantine 1 day after their final test. Central dash: median; light bar: 95% uncertainty interval; dark bar: 50% uncertainty interval. Product of 1,000 infected arriving travellers and 1,000 simulations per scenario.

For single test strategies, a 9-day quarantine with no test reduces the symptomatic entering travellers by 97% (95% UI: 95–99) but asymptomatic entering travellers are only reduced by 80% (95% UI: 4–87), reflecting the difficulty of relying on symptom onset during quarantine. By introducing a PCR test on day 9 and release on day 10, the number of symptomatic entering travellers is reduced by more than 99% (95% UI: 9–100) and asymptomatic entering travellers by 92% (95% UI: 88–97). This difference in detectability of symptomatic and asymptomatic entering travellers, coupled with simulations involving their small absolute numbers, is responsible for driving the wide uncertainty observed in the number of infectious arriving travellers from the US entering the community (Figure 2).

### Effect of reducing the duration of quarantine

To determine if a 14-day quarantine can be replaced by a shorter quarantine with testing, we made RR comparisons to a 14-day quarantine with no test. We see that shorter quarantine periods of 9 days with either one or two rounds of testing may have a similar effect to that of the 14-day quarantine period (RR: 2.0; 95% UI: 1.00–infinity for a test on day 9 with release on day 10; RR: 1.24; 95% UI: 0.53–infinity for a test on day 3 and day 9) (Supplementary Figure S6).

### Pre-flight testing

The impact of pre-flight testing on the number of infectious travellers entering the community was greatest if implemented the day before departure (within 24 h) in scenarios with no post-flight testing, with an RR of 0.69 (95% UI: 0.64–0.73) compared with no testing either before departure or after arrival (Supplementary Figure S4). As quarantine increases in duration, the additional effect of pre-flight testing diminishes.

## Discussion

Here we analysed the effect of different combinations of PCR testing and quarantine times on the number of infectious individuals entering a country, beyond the effect of syndromic screening at the site of departure in the pre-COVID-19 vaccination era. We found that a quarantine period of at least 5 days, combined with a single PCR test on the final day, resulted in a reduction of 89% in the number of infectious individuals entering the community. A 7-day quarantine with a test on the final day can reduce infectious entering travellers by an average of 95%, with a small marginal benefit for additional rounds of testing. In addition, pre-flight testing appears ineffective unless conducted within 24 h of departure; the marginal effect of these tests disappears with increased quarantine duration and post-arrival testing.

We also found that the 14-day quarantine period is highly effective, reducing the number of infectious entering travellers by 99%, on average. Because the 14-day quarantine strategy almost completely eliminates infectious entering travellers, the number of infected entering travellers for other strategies (i.e. 7 or 9 days of quarantine with testing) may represent a two- to fivefold increase in RR. However, the absolute risk of entry is small in these scenarios and so the increase in RR should be interpreted in light of this. The risk stemming from the arrival of infectious travellers will need to be assessed in the context of local infection incidence. For example, six to 11 infectious travellers arriving per week from the EU or US in July 2020 into a community with thousands of live infections (such as that in the UK on 12 June 2020, with a prevalence estimated at 45 infections per 10,000 inhabitants; 95% UI: 24–92) will likely have little impact on control efforts. In contrast, if local infection prevalence is lower (as in the UK on 20 July 2020 with an estimated nine infections per 10,000 inhabitants; 95% UI: 4–18) a higher number of incoming infectious travellers may pose a large risk for seeding outbreaks in the community [33]. Likewise, countries that have pursued policies to eliminate COVID-19 within their borders such as New Zealand may consider any risk of reintroduction as unacceptable [2,34] and therefore continue to pursue policies which minimise the risk as much as practically possible.

We presented the risk from incoming infected travellers as the number of infectious travellers entering the community. To account for the differential residual duration of their infectiousness with the different strategies, we also presented the number of infectious person-days in the community from travellers. While the latter measure indicates an increased effectiveness of longer quarantine, this approach may still underestimate the true effect since the measure only considers that travellers are still infectious, and not how likely transmission is given the viral load. Of note, it is likely that infectiousness is correlated with viral load and declines over the course of infection [35,36]. Hence, with a peak infectivity around the onset of symptoms at ca 5 days, a minimum quarantine period of 7 days is likely to result in the release of fewer infectious travellers, and those who are released at 7 days have less potential for transmission with or without the use of a test.

We assumed that inbound travel volumes were 50% of total traveller movements, as reported by the Civil Aviation Authority, which may not reflect asymmetric patterns of travel. The total number of traveller movements between the UK and both the US and the EU in April and May 2020 were ca 1% of that reported in 2019, indicating that the combination of travel restrictions and suspended airline flights led to a sharp decrease in the number of potentially infected travellers. If travel volumes return to pre-pandemic levels, the likely number of infectious arriving travellers will increase unless prevalence is severely reduced internationally or a greater proportion of the international population is either vaccinated or naturally infected and recovered. In our analysis, we considered a constant air passenger volume and did not consider that shortening of quarantine may lead to an increase in the number of travellers. To address this, we have provided estimates in terms of the number of infectious entering travellers per 10,000 arriving travellers for the given international prevalence.

The work presented here is based on estimates of prevalence, under-ascertainment and travel volumes as of July 2020, as well as the contemporaneous understanding of incubation period, infectiousness and ability to detect an infection by PCR. Multiple studies have found that infectivity peaks around onset of symptoms [28,35,36] and that asymptomatic infections follow similar peak timings but that the detectability by PCR is shorter in duration [37] and lesser in magnitude [25].

We assumed that 70% of travellers with currently symptomatic infections (e.g. with a cough or fever) would be detected or self-report and hence not travel. The remaining 30% are therefore the infectious travellers, who are either symptomatic but undetected, pre-symptomatic or fully asymptomatic. The longer the duration of quarantine, the greater the chance that pre-symptomatic individuals will develop symptoms and self-isolate, further reducing the number of infectious entering travellers. Hence, the primary purpose of quarantine and PCR testing is to reduce possible transmission from asymptomatic travellers who are only detectable by PCR. We also assumed that if individuals subsequently become symptomatic after quarantine, they follow national guidelines to immediately re-enter quarantine and seek an additional test as part of the local test and trace strategy; we assumed that traveller sensitisation is high at this point in the pandemic [38,39]. We do not make any assumptions about the potential for self-isolating infectious travellers to infect their household upon arrival or the resulting onwards transmission. We assumed full adherence to self-isolation, although a first negative test and a long duration of quarantine may reduce adherence to quarantine rules [6,8]. Hence, by assuming perfect adherence, we may overestimate the added benefit of long periods of quarantine in terms of the person-days of infectiousness in the community.

## Conclusions

As the pandemic progresses, public health authorities must carefully balance the need for traveller-targeted interventions that reduce the likelihood of seeding local COVID-19 outbreaks with their social, psychological, financial, and economic costs. While the acceptable number of infected travellers entering the community will depend on the local context of SARS-CoV-2 transmission, we found that for travellers arriving from low prevalence destinations, the absolute risk of infectious entering travellers is likely to be low. Hence, testing and/or quarantine-based strategies may not reduce such risk further, particularly when many infectious arriving travellers are asymptomatic. However, as we have highlighted here, testing is likely the only way to detect asymptomatic infections, and may also detect pre-symptomatic, infectious travellers, leading to earlier isolation. For arriving travellers from countries with ongoing community transmission, quarantine on arrival will limit the risk for onward transmission into the local community in the absence of a safe and effective vaccine against COVID-19. While a 14-day quarantine will likely prevent most transmission from travellers, an 8-day quarantine (with testing on day 7) can capture almost as many infectious individuals in approximately half the time. Testing passengers is resource-intensive but presents a way to either further reduce risks or allow a shorter quarantine at the same level of risk, particularly for travellers arriving from countries with widespread SARS-CoV-2 transmission. Thus, our results contribute to an evidence-based discussion on the benefits and risks of alternative policies on border security regarding SARS-CoV-2 introduction via international air travel.

## Supplementary Data

1538000 Supplement

## References

[1] Chinazzi M Davis JT Ajelli M Gioannini C Litvinova M Merler S The effect of travel restrictions on the spread of the 2019 novel coronavirus (COVID-19) outbreak. Science. 2020;368(6489):395-400. 10.1126/science.aba9757 32144116 PMC7164386

[2] Han E Tan MMJ Turk E Sridhar D Leung GM Shibuya K Lessons learnt from easing COVID-19 restrictions: an analysis of countries and regions in Asia Pacific and Europe. Lancet. 2020;396(10261):1525-34. 10.1016/S0140-6736(20)32007-9 32979936 PMC7515628

[3] Department of Health and Social Care and Department for Transport. Coronavirus (COVID-19): how to self-isolate when you travel to the UK. London: United Kingdom Government. [Accessed: 20 Jul 2020]. Available from: https://www.gov.uk/government/publications/coronavirus-covid-19-how-to-self-isolate-when-you-travel-to-the-uk/coronavirus-covid-19-how-to-self-isolate-when-you-travel-to-the-uk

[4] Li Q Guan X Wu P Wang X Zhou L Tong Y Early transmission dynamics in Wuhan, China, of novel coronavirus-infected pneumonia. N Engl J Med. 2020;382(13):1199-207. 10.1056/NEJMoa2001316 31995857 PMC7121484

[5] World Health Organisation (WHO). Operational considerations for case management of COVID-19 in health facility and community. Report No.: WHO/2019-nCoV/HCF_operations/2020.1. Geneva: WHO; 2020. Available from: https://www.who.int/publications/i/item/10665-331492

[6] Brooks SK Webster RK Smith LE Woodland L Wessely S Greenberg N The psychological impact of quarantine and how to reduce it: rapid review of the evidence. Lancet. 2020;395(10227):912-20. 10.1016/S0140-6736(20)30460-8 32112714 PMC7158942

[7] Pfefferbaum B North CS. Mental health and the covid-19 pandemic. N Engl J Med. 2020;383(6):510-2. 10.1056/NEJMp2008017 32283003

[8] Webster RK Brooks SK Smith LE Woodland L Wessely S Rubin GJ. How to improve adherence with quarantine: rapid review of the evidence. Public Health. 2020;182:163-9. 10.1016/j.puhe.2020.03.007 32334182 PMC7194967

[9] United States Food and Drug Administration (FDA). Quest SARS-CoV-2 rRT-PCR (Quest Diagnostics Infectious Disease, Inc.) - Manufacturer Instructions/Package Insert. Silver Spring: FDA. [Accessed: 16 Jul 2020]. Available from: https://www.fda.gov/media/136231/

[10] Nikkei Staff Writers. Japan set to lift quarantines for business travelers in summer. Nikkei Asian Review. [Accessed: 6 Jul 2020]. Available from: https://asia.nikkei.com/Business/Travel-Leisure/Japan-set-to-lift-quarantines-for-business-travelers-in-summer

[11] Civil Aviation Authority. Airport data. 2019 07 | London: Civil Aviation Authority. [Accessed: 20 Jul 2020]. Available from: https://www.caa.co.uk/Data-and-analysis/UK-aviation-market/Airports/Datasets/UK-Airport-data/Airport-data-2019-07

[12] Civil Aviation Authority. Airport data. 2020 05. London: Civil Aviation Authority. [Accessed: 4 Jul 2020]. Available from: https://www.caa.co.uk/Data-and-analysis/UK-aviation-market/Airports/Datasets/UK-Airport-data/Airport-data-2020-05

[13] Russell TW Golding N Hellewell J Abbott S Wright L Pearson CAB Reconstructing the early global dynamics of under-ascertained COVID-19 cases and infections. BMC Med. 2020;18(1):332. 10.1186/s12916-020-01790-9 33087179 PMC7577796

[14] Russell TW Wu JT Clifford S Edmunds WJ Kucharski AJ Jit M Effect of internationally imported cases on internal spread of COVID-19: a mathematical modelling study. Lancet Public Health. 2021;6(1):e12-20. 10.1016/S2468-2667(20)30263-2 33301722 PMC7801817

[15] Buitrago-Garcia D Egli-Gany D Counotte MJ Hossmann S Imeri H Ipekci AM Occurrence and transmission potential of asymptomatic and presymptomatic SARS-CoV-2 infections: A living systematic review and meta-analysis. PLoS Med. 2020;17(9):e1003346. 10.1371/journal.pmed.1003346 32960881 PMC7508369

[16] United Kingdom Government. Coronavirus (COVID-19): getting tested. London: gov.uk. [Accessed: 12 Jun 2020]. Available from: https://www.gov.uk/guidance/coronavirus-covid-19-getting-tested

[17] Quilty BJ Clifford S Flasche S Eggo RM CMMID nCoV working group. Effectiveness of airport screening at detecting travellers infected with novel coronavirus (2019-nCoV). Euro Surveill. 2020;25(5):2000080. 10.2807/1560-7917.ES.2020.25.5.2000080 32046816 PMC7014668

[18] Gostic K Gomez AC Mummah RO Kucharski AJ Lloyd-Smith JO. Estimated effectiveness of symptom and risk screening to prevent the spread of COVID-19. eLife. 2020;9:e55570. 10.7554/eLife.55570 32091395 PMC7060038

[19] Gostic K. Estimated effectiveness of symptom and risk screening to prevent the spread of COVID-19. San Francisco: Github. [Accessed: 16 Jul 2020]. Available from: https://kgostic.github.io/traveller_screening 10.7554/eLife.55570 PMC7060038 32091395

[20] International Air Transport Association (IATA). Criteria for COVID-19 testing in the air travel process. Geneva: IATA. [Accessed: 4 Jul 2020]. Available from: https://www.iata.org/en/pressroom/pr/2020-06-16-02/

[21] Xinhua Global Service. [Beijing Railway Department: Implementing ticket purchase restrictions for high-risk groups and leaving Beijing with a negative certificate of nucleic acid test within 7 days]. Xinhuanet. [Accessed: 6 Jul 2020]. Chinese. Available from: http://www.xinhuanet.com/politics/2020-06/23/c_1126152337.htm

[22] National Health Service (NHS). Main symptoms of coronavirus (COVID-19). London: NHS. [Accessed: 20 Jul 2020]. Available from: https://www.nhs.uk/conditions/coronavirus-covid-19/symptoms/main-symptoms

[23] Kucirka LM Lauer SA Laeyendecker O Boon D Lessler J. Variation in false-negative rate of reverse transcriptase polymerase chain reaction-based SARS-CoV-2 tests by time since exposure. Ann Intern Med. 2020;173(4):262-7. 10.7326/M20-1495 32422057 PMC7240870

[24] Grassly N, Pons Salort M, Parker E, White P, Ainslie K, Baguelin M, et al. Report 16: Role of testing in COVID-19 control. London: Imperial College London; 2020. Available from: https://spiral.imperial.ac.uk:8443/handle/10044/1/78439

[25] Van Vinh Chau N Lam VT Dung NT Yen LM Minh NNQ Hung LM The natural history and transmission potential of asymptomatic SARS-CoV-2 infection. Clin Infect Dis. 2020;71(10):2679. 10.1093/cid/ciaa711 32497212 PMC7314145

[26] Lauer SA Grantz KH Bi Q Jones FK Zheng Q Meredith HR The incubation period of coronavirus disease 2019 (COVID-19) from publicly reported confirmed cases: estimation and application. Ann Intern Med. 2020;172(9):577-82. 10.7326/M20-0504 32150748 PMC7081172

[27] Davies NG Klepac P Liu Y Prem K Jit M CMMID COVID-19 working group Eggo RM. Age-dependent effects in the transmission and control of COVID-19 epidemics. Nat Med. 2020;26(8):1205-11. 10.1038/s41591-020-0962-9 32546824

[28] He X Lau EHY Wu P Deng X Wang J Hao X Temporal dynamics in viral shedding and transmissibility of COVID-19. Nat Med. 2020;26(5):672-5. 10.1038/s41591-020-0869-5 32296168

[29] Ashcroft P Huisman JS Lehtinen S Bouman JA Althaus CL Regoes RR COVID-19 infectivity profile correction. Swiss Med Wkly. 2020;150:w20336. 32757177 10.4414/smw.2020.20336

[30] Wölfel R Corman VM Guggemos W Seilmaier M Zange S Müller MA Virological assessment of hospitalized patients with COVID-2019. Nature. 2020;581(7809):465-9. 10.1038/s41586-020-2196-x 32235945

[31] Byrne AW McEvoy D Collins AB Hunt K Casey M Barber A Inferred duration of infectious period of SARS-CoV-2: rapid scoping review and analysis of available evidence for asymptomatic and symptomatic COVID-19 cases. BMJ Open. 2020;10(8):e039856. 10.1136/bmjopen-2020-039856 32759252 PMC7409948

[32] R Core Team. R: A Language and Environment for Statistical Computing. Vienna: R Foundation for Statistical Computing; 2020. Available from: https://www.R-project.org

[33] Russell TW Wu JT Clifford S Edmunds WJ Kucharski AJ Jit M Centre for the Mathematical Modelling of Infectious Diseases COVID-19 working group. Effect of internationally imported cases on internal spread of COVID-19: a mathematical modelling study. Lancet Public Health. 2021;6(1):e12-20. 10.1016/S2468-2667(20)30263-2 33301722 PMC7801817

[34] Baker MG Kvalsvig A Verrall AJ. New Zealand’s COVID-19 elimination strategy. Med J Aust. 2020;213(5):198-200.e1. 10.5694/mja2.50735 32789868 PMC7436486

[35] Cevik M Tate M Lloyd O Maraolo AE Schafers J Ho A. SARS-CoV-2, SARS-CoV, and MERS-CoV viral load dynamics, duration of viral shedding, and infectiousness: a systematic review and meta-analysis. Lancet Microbe. 2021;2(1):e13-22. 10.1016/S2666-5247(20)30172-5 33521734 PMC7837230

[36] Kissler SM Fauver JR Mack C Olesen SW Tai C Shiue KY Viral dynamics of acute SARS-CoV-2 infection and applications to diagnostic and public health strategies. PLoS Biol. 2021;19(7):e3001333. 10.1371/journal.pbio.3001333 34252080 PMC8297933

[37] Quilty BJ Clifford S Hellewell J Russell TW Kucharski AJ Flasche S Quarantine and testing strategies in contact tracing for SARS-CoV-2: a modelling study. Lancet Public Health. 2021;6(3):e175-83. 10.1016/S2468-2667(20)30308-X 33484644 PMC7826085

[38] Hellewell J Abbott S Gimma A Bosse NI Jarvis CI Russell TW Feasibility of controlling COVID-19 outbreaks by isolation of cases and contacts. Lancet Glob Health. 2020;8(4):e488-96. 10.1016/S2214-109X(20)30074-7 32119825 PMC7097845

[39] Clifford S Pearson CAB Klepac P Van Zandvoort K Quilty BJ Eggo RM Effectiveness of interventions targeting air travellers for delaying local outbreaks of SARS-CoV-2. J Travel Med. 2020;27(5):taaa068. 10.1093/jtm/taaa068 32384159 PMC7239177

[40] Pya N Wood SN. Shape constrained additive models. Stat Comput. 2015;25(3):543-59. 10.1007/s11222-013-9448-7

[41] Devroye L. General Principles in Random Variate Generation. Non-Uniform Random Variate Generation. Springer: New York, NY; 1986. 10.1007/978-1-4613-8643-8_2

