# Supplementary material for: Strategies to reduce the risk of SARS-CoV-2 importation from international travellers: modelling estimations for the United Kingdom, July 2020
Source: Euro Surveill. 2021 Sep 30;26(39):2001440. doi: 10.2807/1560-7917.ES.2021.26.39.2001440 (PMC8485583; doi:10.2807/1560-7917.ES.2021.26.39.2001440)
Supplement: Supplement [file 20-01440_QUILTY_Supplement.pdf]

Strategies to reduce the risk of SARS-CoV-2 importation from international travellers, modelling estimations, July 2020 - Supplementary Appendix

This supplementary material is hosted by Eurosurveillance as supporting information alongside the article “Strategies to reduce the risk of SARS-CoV-2 importation from international travellers, modelling estimations, July 2020” on behalf of the authors, who remain responsible for the accuracy and appropriateness of the content. The same standards for ethics, copyright, attributions and permissions as for the article apply. Supplements are not edited by Eurosurveillance and the journal is not responsible for the maintenance of any links or email addresses provided therein.

Number of infected travellers

Civil Aviation Authority data for April and May 2020 indicates that traveller volume was approximately 99% lower compared to the same period in 2019 (Table S1). The traveller volumes in July 2020 are therefore assumed to be approximately 1% of those in July 2019.

Table S1: Traveller movements in June 2019 and year on year change for May 2020 compared to May 2019 between UK airports, and airports in the European Union (EU) and United States of America (USA). Source: Civil Aviation Authority Tables 10.1 and 12.1 for July 2019 [1], May 2019 [1] and May 2020 [2].

	EU	USA	Source
Total traveller volume July 2019	18,186,680	2,249,856	[1]
Year-on-year change for April and May 2020 compared to April and May 2019, %	-99%	-99%	[2] EU: Table S10.1 USA: Table S12.1
Calculated total traveller volume July 2020 using May year-on-year change, n	181,187	22,499	[1,2] EU: Table S10.1 USA: Table S12.1
Duration of typical flight (hours)	2	8	Assumed
Prevalence of SARS-CoV-2 on 20 July 2020	2.8 per 10,000	40.0 per 10,000	[3]
Number of infected individuals intending to travel in a given week. Median and 95% interval from 1000 simulations.	Symptomatic: 4 (1, 10) Asymptomatic: 1 (0, 5)	Symptomatic: 8 (2, 21) Asymptomatic: 2 (0, 10)	Proportion asymptomatic derived from [4]

We assume that the observed weekly travel volume, here, W, is those who have not been screened out or self-selected out based on onset of symptoms, i.e. the sum of the number of uninfected, asymptomatic, and those ever-symptomatic travellers not currently symptomatic The total number of intending travellers, W', is W, plus those who do not travel, δW. We

calculate W' as follows. First, sample $W \sim Bin(p = 7/30, \lceil n/2 \rceil)$. For α, the proportion of infections which are asymptomatic, π, the prevalence at the travel origin, ξ, the proportion of ever-symptomatic cases who are symptomatic at intended time of departure, and ρ, the proportion of currently symptomatic travellers prevented from boarding, δW is distributed according to a negative binomial distribution with size W and $p = 1 - \pi(1 - \alpha)\rho\xi$. ξis estimated by sampling a large number of ever-symptomatic travellers, along with flight departure times and symptomatic periods and determining which proportion are symptomatic at time of intended departure.

The number of uninfected travellers, S, is then $S \sim Bin(1 - \pi, W + \delta W)$; the number of asymptomatic infected travellers is $I_a \sim Bin(\alpha, W + \delta W - S)$; the number of travellers symptomatic at time of departure is $I_s \sim Bin\left(\xi, W + \delta W - S - I_a\right)$and the number of ever-symptomatic travellers who are permitted to travel is therefore $W + \delta W - S - I_a - I_s$ and is composed of those who are not yet symptomatic, those who are post-symptomatic, and those who are symptomatic but not detected by syndromic screening.

Risk mitigation strategies

At maximum stringency, the 14 day quarantine period aims to ensure that even a traveller who was infected just before or during the flight would likely spend their whole infectious period in quarantine and thereby not infect others. The moderately stringent strategy, on the other hand, aims to ensure that travellers spend a sufficient amount of time in quarantine to allow for the development of symptoms and probability of a positive PCR test leading to isolation for those infected. These strategies would, however, risk that some asymptomatically infected travellers (that is, infected travellers who will never display symptoms) will enter the community before the end of their infectious period.

Table S2 - Strategies for risk mitigation. Where one of the described lines contains “or”, we consider all combinations contained within. For all levels of stringency we consider scenarios with the following pre-flight PCR policies: no pre-flight testing, pre-flight testing within 1 day of departure, within 4 days of departure, or within 1 week of departure.

**Stringency of screening policy***	**Description of screening policy**
Low	01. No mandatory quarantine on arrival, and<br>02. Either no post-flight testing, *or* a single PCR test on arrival.<br>03. Release immediately after arrival (no test) or on receipt of negative result (test).<br>*We consider a no-quarantine, no-testing scenario as the primary baseline for comparison.*
Moderate	01. Mandatory 3, 5 or 7 days quarantine on arrival, and<br>02. Either no post-flight testing *or* a single PCR test at end of mandatory quarantine<br>03. Release at end of mandatory quarantine period (no test) *or* on receipt of negative test at end of mandatory quarantine period.
High	01. Mandatory quarantine on arrival, and<br>02. A first PCR test 0, 1 or 2 days after arrival, and

	03. A second PCR test either 2, 4 or 6 days after the first<br>04. Release after two negative post-arrival results or 14 days after earliest positive post-arrival test.
Maximum	01. Mandatory 14 days quarantine on arrival<br>02. Either no post-flight testing *or* a single PCR test at end of mandatory quarantine<br>03. Release at end of mandatory quarantine period (no test) *or* on receipt of negative test at end of mandatory quarantine period.

* In all scenarios we assumed that syndromic screening is implemented at the departure airport, hence low stringency rather than no stringency.

Detection model

The time-varying PCR sensitivity is modelled as a function of the time since an individual's exposure (Figure 1, Kucirka et al. 2020 [5]) and derived by fitting a Generalised Additive Model (GAM) with a Binomial likelihood and penalised B-spline basis (P-spline) [6], to the data collected by Kucirka et al. (2020) [5]. We shift the observations, as they have, by an incubation period of 5 days [7], and augment by a pseudo-negative test on day 0 for each of the constituent data sets.

Table S3 - Values of parameters in simulation of travellers' infection histories and PCR testing. Gamma distributions are parameterised in terms of a mean and variance, $\Gamma(\mu, \sigma^2)$, and these are converted to shape and rate parameters via moment matching. Where quantiles are given but no distribution described, the parameter is derived from other distributions in the table and has no closed-form.

Model parameter	Description	Value	Source
Incubation period (days)	Time from exposure to onset of symptoms.	$\Gamma\left(\mu = 5.5, \sigma^2 = 6.5\right)$<br>Median: 5.1 days<br>IQR: (3.6, 6.9) days<br>95%: (1.7, 11.5) days	Derived from quantile matching with Median: 5.1 days, 97.5%: 11.5 days [7]
Time to infectiousness (symptomatic cases)	Time after exposure (and before onset of symptoms) from which pre-symptomatic transmission can occur.	Median: 3.4 days<br>IQR: (2.3, 4.9) days<br>95%: (0.9, 8.6) days	Derived from [8]
Infectious period (symptomatic cases, days)	Duration of period in which case is able to infect others	Median: 7.1 days<br>IQR: (5.7, 8.5) days<br>95%: (2.5, 11.6) days	Derived from [9]
Symptomatic period (symptomatic cases, days)	Time after onset of symptoms until no longer symptomatic	$\Gamma(\mu = 9.1, \sigma^2 = 14.7)$<br>Median: 8.6 days<br>IQR: (6.3, 11.3) days<br>95%: (3.2, 18.0) days	Derivation from [10] based on moment matching distributions in [11]

Fraction of currently symptomatic travellers, ξ	Proportion of ever-symptomatic infections symptomatic at intended departure time	0.44	Derived from simulation of travellers
Syndromic screening detection rate, ρ	Proportion of symptomatic individuals intending to travel who are either screened out at point of departure or self-select out of travelling	0.7	Derived from [12]
Infectious period (asymptomatic cases, days)	Duration of period in which case is able to infect others	$\Gamma\left(\mu = 6, \sigma^2 = 12\right)$<br>Median: 5.3 days<br>IQR: (3.5, 7.8) days<br>95%: (1.2, 14.4) days	Assumption based on [13]
PCR sensitivity for symptomatic infections (Figure S1A)	Probability of testing PCR positive *t* days after infection, if infection is symptomatic	$P(t)$	Penalised B-spline fit to data in [5]
PCR specificity	Probability of a negative PCR test given no infection with SARS-CoV-2.	1	Assumption consistent with [14]
Asymptomatic fraction, α	Proportion of infections which are asymptomatic.	$Beta(1.9,\ 6.3)$<br>Median: 0.21<br>IQR: (0.12, 0.32)<br>95%: (0.03, 0.55)	Derived from quantile matching, 95%: (0.03, 0.55) [4]
PCR sensitivity for asymptomatic infections	Probability of testing PCR positive t days after infection, if infection is asymptomatic	$0.62 * P(t)$	Scaling factor derived from [15]

According to He et al. (2020) infectiousness of symptomatic cases begins up to 12.3 days (95%: (5.9, 17) days) prior to the onset of symptoms and peaks at onset of symptoms (0 days, 95%: -0.9, 0.9 days) [8,16]. We sampled this pre-symptomatic infectious period duration to derive the time from exposure to infectiousness by matching the quantiles of the distribution of time to onset of symptoms to the quantiles of the distribution of infectiousness lead times for each traveller, preserving order, ensuring that no time to infectiousness occurs before exposure. The duration of the infectious period for symptomatic cases was derived from the data of Wölfel et al. (2020) [9] by fitting a Binomial GAM with P-splines to determine the probability of no longer being infectious as a function of days since onset of symptoms. The time to non-infectiousness is sampled from the fitted GAM, which has range (0,1), by the inverse transform method [17].

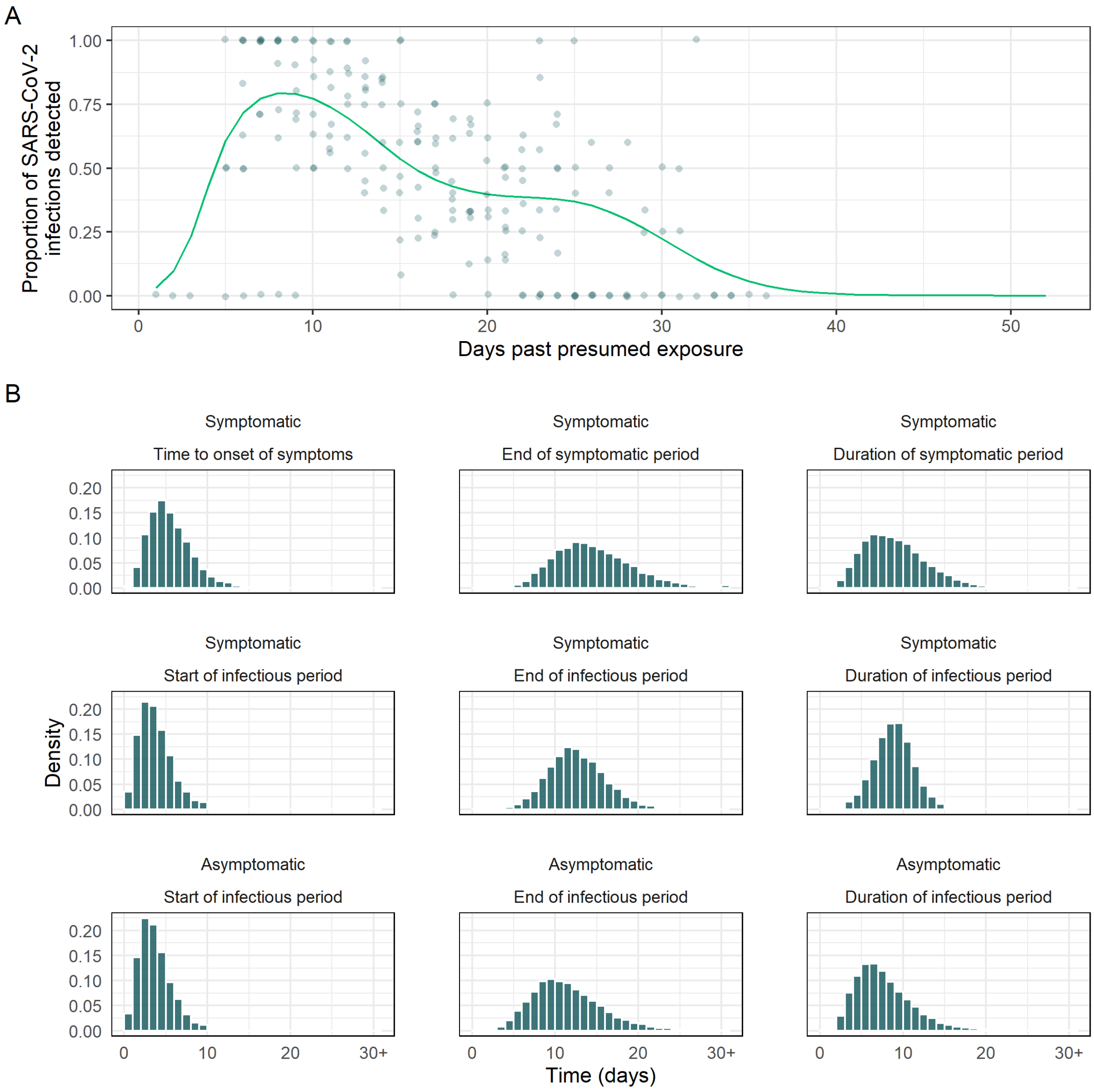

Figure S1 - A. Traveller PCR sensitivity curves, obtained by fitting a Binomial GAM to the data collated in Kucirka et al. (2020) [5] The mean fit is used as the time-varying sensitivity function, $P(t)$, and hence no uncertainty is shown in the figure. B. Distributions of times to clinically relevant events, namely time from exposure to start and end, and duration, of symptoms for symptomatic infections (dark green), and infectiousness for both symptomatic and asymptomatic (light green) infections. Times greater than 30 days are collapsed to a single “30+” bin.

Results

As a baseline for comparison, we use the lowest stringency scenario considered: 70% of currently symptomatic travellers are prevented from boarding, but no quarantine or testing is conducted. In this scenario, between 2 and 12 (EU), and 3 and 24 (USA) infectious travellers would enter the community (Figure S2A, low, no testing). By introducing a mandatory quarantine period of 7 days, this can be reduced to 0 to 3 infectious persons per week from the EU and 0 to 4 from the USA (Figure S2A, Mod.), preventing approximately 80% of

travellers from entering the community while being infectious (Rate Ratios, median and 95% UI: EU: 0.18 (0.00, 0.42), USA: 0.18 (0.10, 0.27)). A mandatory quarantine period of 14 days resulted in 0 to 1 infectious entries per week each from the EU and USA (Figure S2A, Max.), an almost completely effective reduction (RR: EU: 0.00 (0.00, 0.01), USA: 0.01 (0.00, 0.04)).

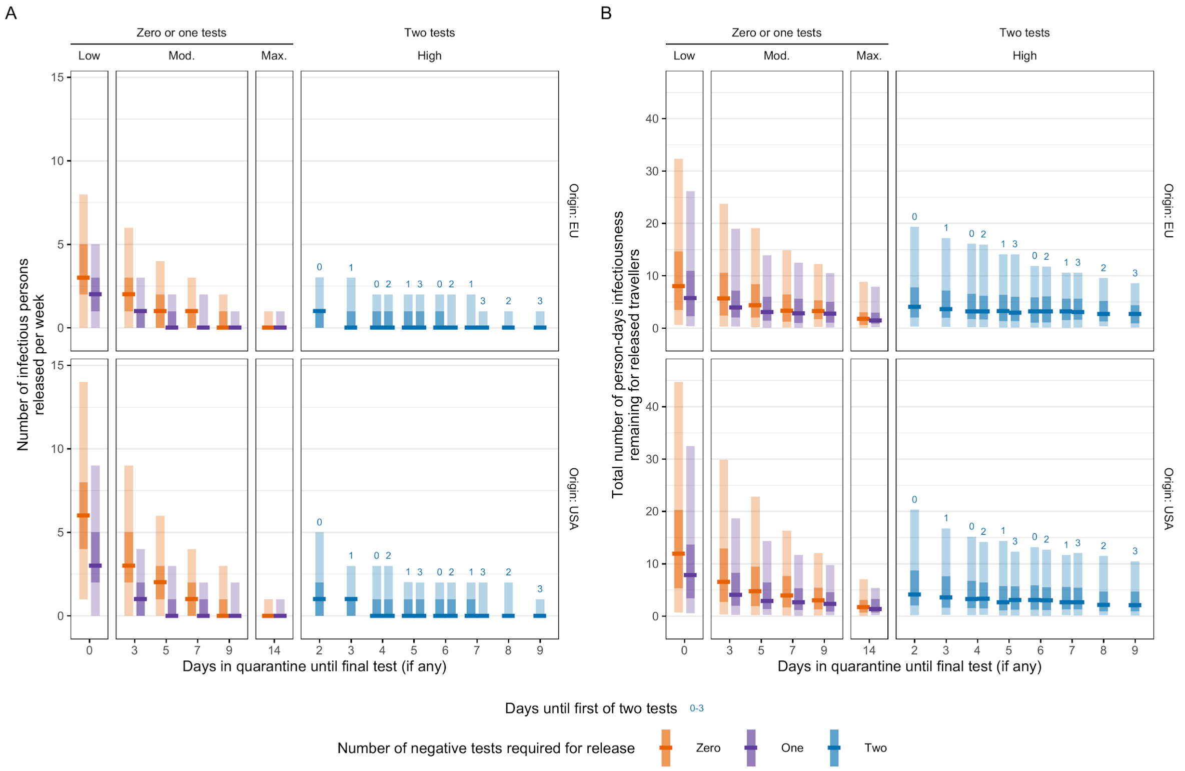

Figure S2: A. Expected number of infectious and pre-infectious persons free to enter the UK from the EU and USA based on observed travel volumes in each of the scenarios and how long they spend in quarantine before release, with no pre-flight testing. B. Total person-days of infectiousness remaining after release, based on observed travel volumes. We assume that test results are delayed by 1 day and hence persons leave quarantine 1 day after their final test. Central bar = median; light bar = 95% uncertainty interval; dark bar = 50% uncertainty interval.

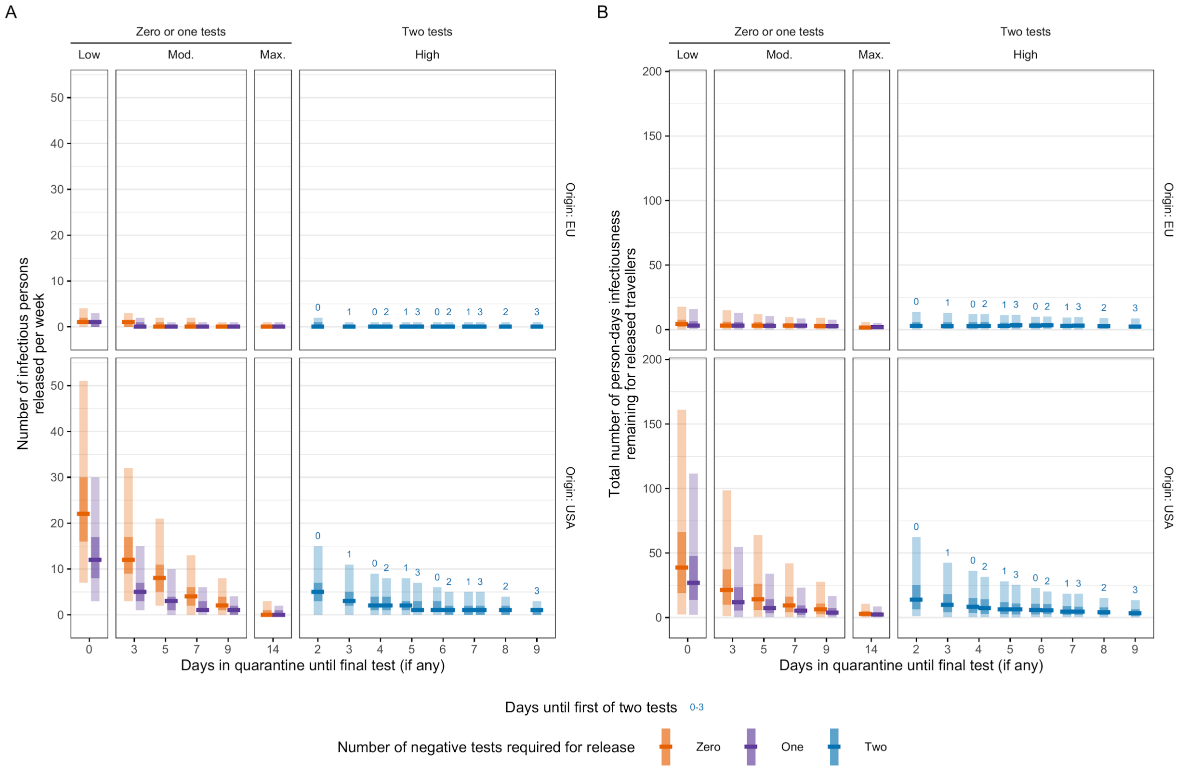

Figure S3 - As for Figure S1 but per 10,000 travellers rather than observed flight volumes.

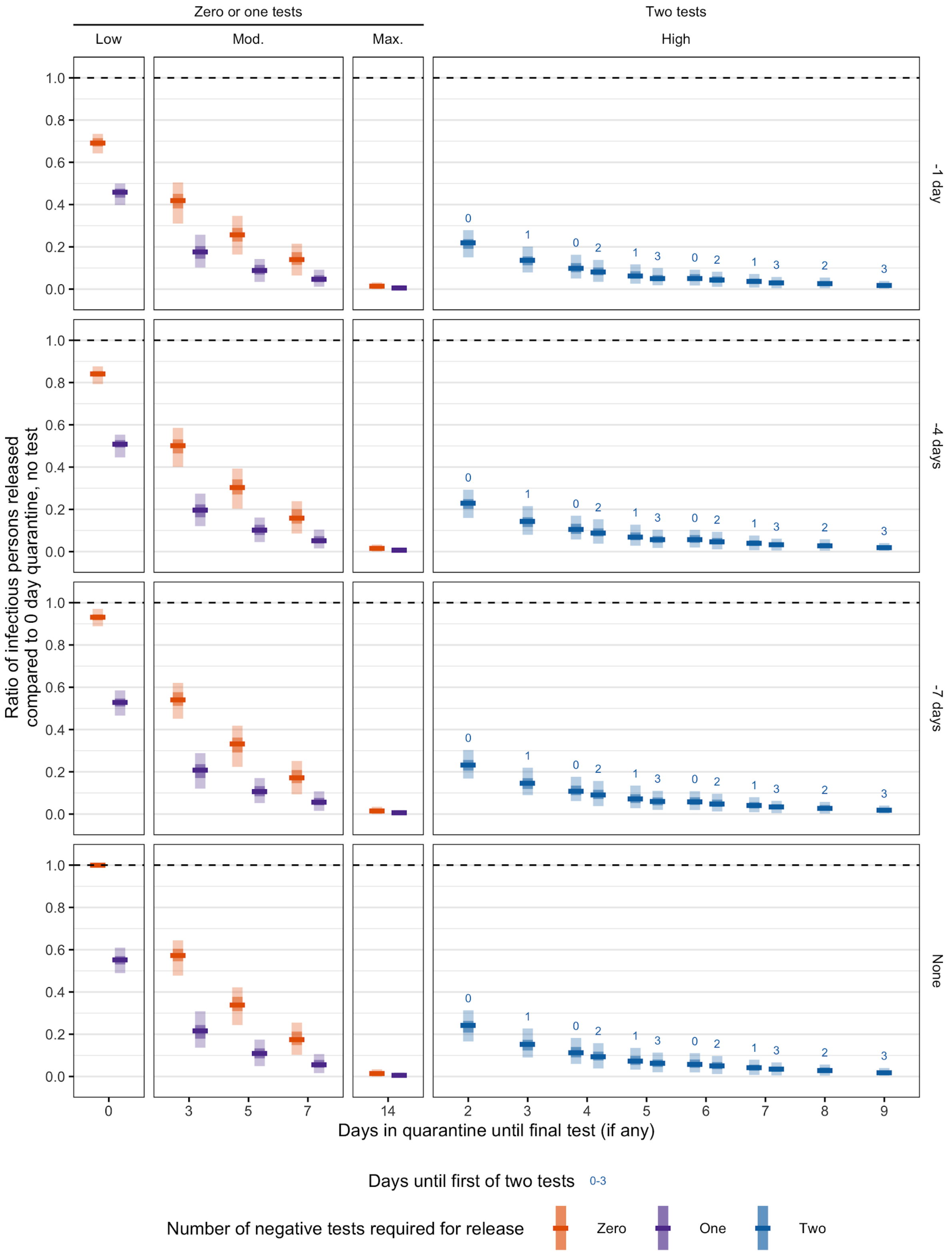

Figure S4 - Per-infected traveller reduction in risk given by each strategy in comparison to a baseline of a 0 day quarantine on arrival with no testing, considering either no pre-flight testing, or pre-flight testing 1, 4 or 7 days prior to departure. We assume that test results are delayed by 1 day and hence persons leave quarantine 1 day after their second test. Central bar = median; light bar = 95% uncertainty interval; dark bar = 50% uncertainty interval. Product of 1000 infected arrivals and 1000 simulations per scenario.Persons showing symptoms at departure were assumed to be prevented from travel, and post-infectious persons were assumed to not carry any

risk of seeding transmission. We assume that test results are delayed by 1 day and hence persons leave self-isolation 1 day after their final test. Central bar = median; light bar = 95% uncertainty interval; dark bar = 50% uncertainty interval.

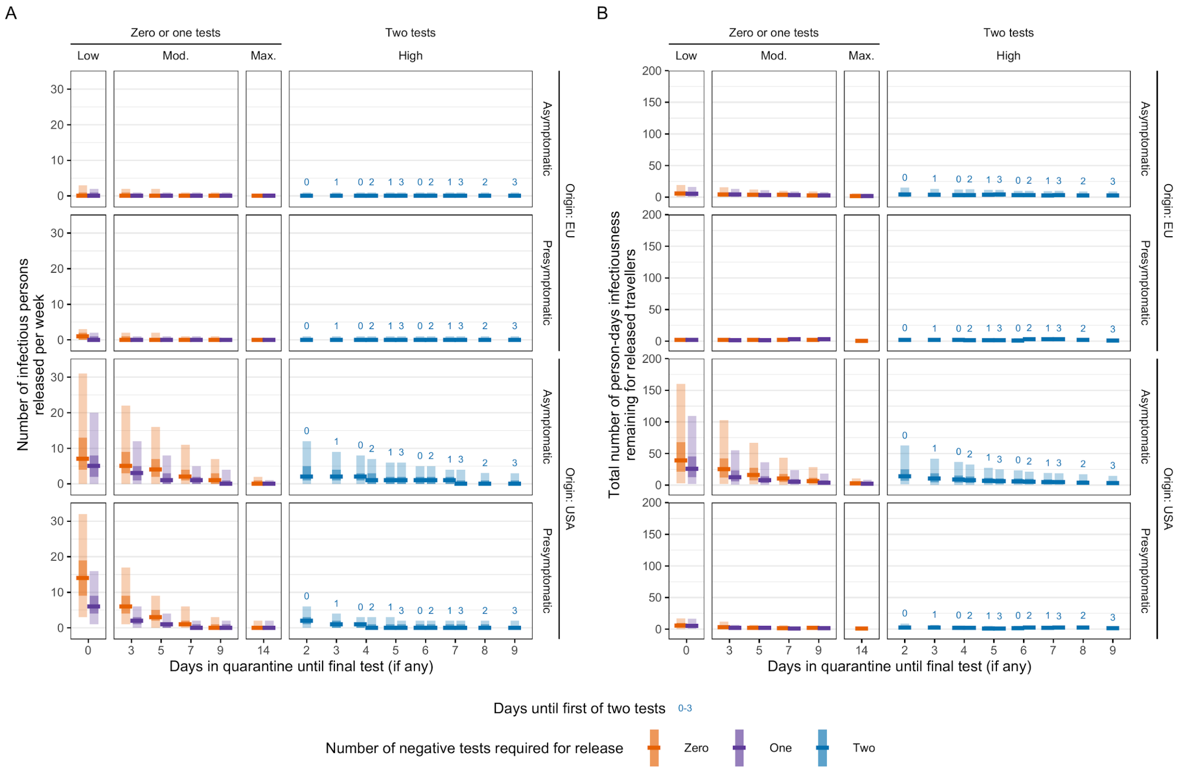

Figure S5 - As for Figure S3 but stratified on whether infection is asymptomatic or presymptomatic.

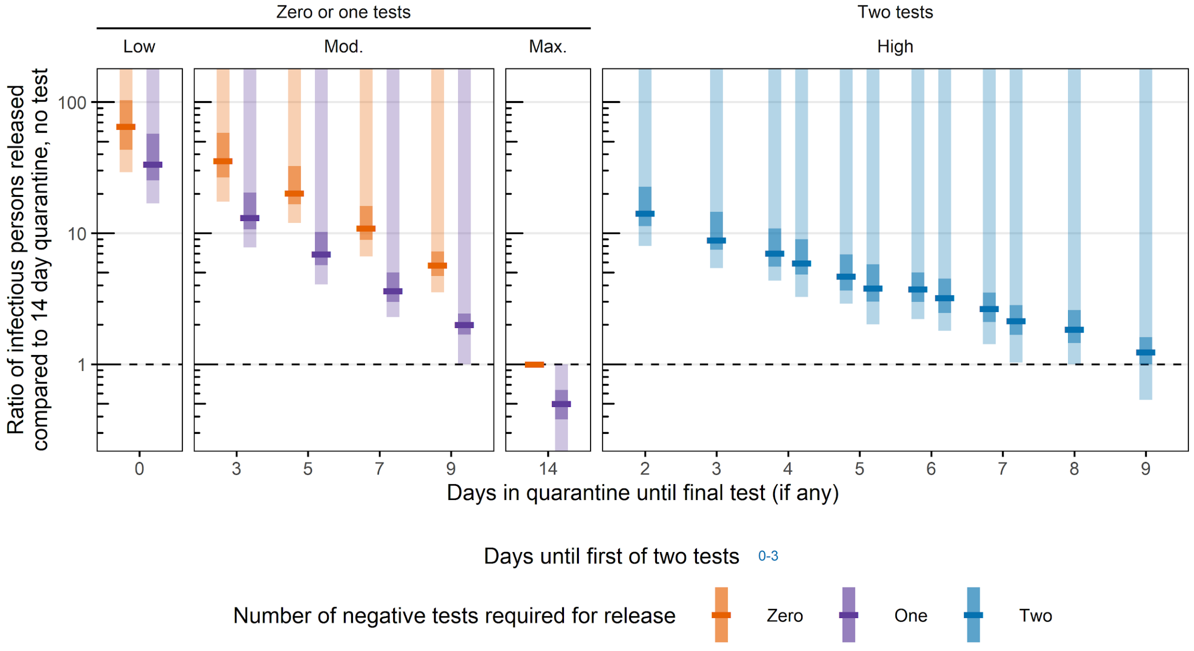

Figure S6 - Per-infected traveller reduction in risk given by each strategy in comparison to a baseline of a 14 day quarantine on arrival with no testing. We assume that test results are delayed by 1 day and hence persons leave quarantine 1 day after their final test. Central bar = median; light bar = 95% uncertainty interval; dark bar = 50% uncertainty interval. Product of 1000 infected arrivals and 1000 simulations per scenario.

[1] Airport data 2019 07 | UK Civil Aviation Authority n.d. https://www.caa.co.uk/Data-and-analysis/UK-aviation-market/Airports/Datasets/UK-Airport-data/Airport-data-2019-07/ (accessed July 20, 2020).

[2] Airport data 2020 05 | UK Civil Aviation Authority n.d. https://www.caa.co.uk/Data-and-analysis/UK-aviation-market/Airports/Datasets/UK-Airport-data/Airport-data-2020-05/ (accessed July 4, 2020).

[3] Russell TW, Joel Hellewell SA, Golding N, Gibbs H, Jarvi CI, Kevin van Zandvoort, et al. Using a delay-adjusted case fatality ratio to estimate under-reporting. 2020.

[4] Buitrago-Garcia DC, Egli-Gany D, Counotte MJ, Hossmann S, Imeri H, Ipekci AM, et al. The role of asymptomatic SARS-CoV-2 infections: rapid living systematic review and meta-analysis. Epidemiology 2020. https://doi.org/10.1101/2020.04.25.20079103.

[5] Kucirka LM, Lauer SA, Laeyendecker O, Boon D, Lessler J. Variation in False-Negative Rate of Reverse Transcriptase Polymerase Chain Reaction-Based SARS-CoV-2 Tests by Time Since Exposure. Ann Intern Med 2020. https://doi.org/10.7326/M20-1495.

[6] Pya N, Wood SN. Shape constrained additive models. Stat Comput 2015;25:543–59. https://doi.org/10.1007/s11222-013-9448-7.

[7] Lauer SA, Grantz KH, Bi Q, Jones FK, Zheng Q, Meredith HR, et al. The Incubation Period of Coronavirus Disease 2019 (COVID-19) From Publicly Reported Confirmed Cases: Estimation and Application. Ann Intern Med 2020;172:577–82. https://doi.org/10.7326/M20-0504.

[8] He X, Lau EHY, Wu P, Deng X, Wang J, Hao X, et al. Temporal dynamics in viral shedding and transmissibility of COVID-19. Nat Med 2020;26:672–5. https://doi.org/10.1038/s41591-020-0869-5.

[9] Wölfel R, Corman VM, Guggemos W, Seilmaier M, Zange S, Müller MA, et al. Virological assessment of hospitalized patients with COVID-2019. Nature 2020;581:465–9. https://doi.org/10.1038/s41586-020-2196-x.

[10] Quilty BJ, Clifford S, Flasche S, Eggo RM, CMMID nCoV working group. Effectiveness of airport screening at detecting travellers infected with novel coronavirus (2019-nCoV). Euro Surveill 2020;25. https://doi.org/10.2807/1560-7917.ES.2020.25.5.2000080.

[11] Li Q, Guan X, Wu P, Wang X, Zhou L, Tong Y, et al. Early Transmission Dynamics in Wuhan, China, of Novel Coronavirus-Infected Pneumonia. N Engl J Med 2020;382:1199–207. https://doi.org/10.1056/NEJMoa2001316.

[12] Gostic K, Gomez AC, Mummah RO, Kucharski AJ, Lloyd-Smith JO. Estimated effectiveness of symptom and risk screening to prevent the spread of COVID-19. Elife 2020;9. https://doi.org/10.7554/eLife.55570.

[13] Byrne AW, McEvoy D, Collins A, Hunt K, Casey M, Barber A, et al. Inferred duration of infectious period of SARS-CoV-2: rapid scoping review and analysis of available evidence for asymptomatic and symptomatic COVID-19 cases. Epidemiology 2020. https://doi.org/10.1101/2020.04.25.20079889.

[14] Grassly N, Pons Salort M, Parker E, White P, Ainslie K, Baguelin M, et al. Report 16: Role of testing in COVID-19 control 2020. https://doi.org/10.25561/78439.

[15] Chau NVV, Thanh Lam V, Thanh Dung N, Yen LM, Minh NNQ, Hung LM, et al. The natural history and transmission potential of asymptomatic SARS-CoV-2 infection. Clin Infect Dis 2020. https://doi.org/10.1093/cid/ciaa711.

[16] Ashcroft P, Huisman JS, Lehtinen S, Bouman JA, Althaus CL, Regoes RR, et al. COVID-19 infectivity profile correction. arXiv [q-bioPE] 2020.

[17] Devroye L. General Principles in Random Variate Generation. Non-Uniform Random Variate Generation 1986:27–82. https://doi.org/10.1007/978-1-4613-8643-8_2.
